# A primer on infrarenal abdominal aortic aneurysms

**DOI:** 10.12688/f1000research.11860.1

**Published:** 2017-08-23

**Authors:** Norman R Hertzer

**Affiliations:** 1 Department of Vascular Surgery, Cleveland Clinic Emeritus Office, Beechwood, OH, USA

**Keywords:** abdominal aortic aneurysm, endovascular repair, rupture, open repair

## Abstract

Ruptured abdominal aortic aneurysms have an alarmingly high mortality rate that often exceeds 50%, even when patients survive long enough to be transported to hospitals. Historical data have shown that ruptures are especially likely to occur with aneurysms measuring ≥6 cm in diameter, but there are so many exceptions to this that several randomized clinical trials have been done in an attempt to determine whether smaller aneurysms should be repaired electively as soon as they are discovered. More recently, further trials have been conducted in order to compare the relative benefits and disadvantages of modern endovascular aneurysm repair to those of traditional open surgery. This review summarizes current evidence from randomized trials and large population-based datasets regarding two questions that are uppermost in the mind of virtually every patient who is found to have an abdominal aortic aneurysm. Should it be fixed? What are the risks?

## Introduction

**Figure 1.**
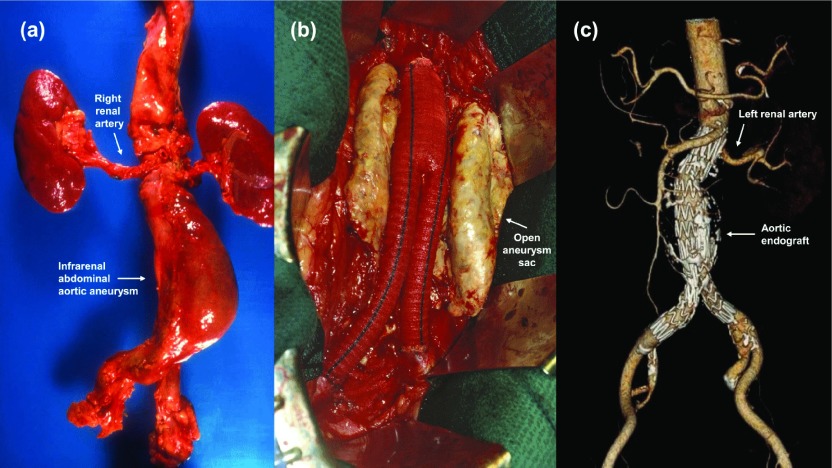
Images of infrarenal abdominal aortic aneurysms. ( **a**) Autopsy specimen showing the relationship of an intact infrarenal aneurysm to the renal arteries. ( **b**) Operative photograph during transabdominal open repair with a knitted bifurcation graft. ( **c**) Three-dimensional computed tomogram after transfemoral endovascular repair in another patient.

Non-septic abdominal aortic aneurysms (AAAs) are caused by weakening and fragmentation of the internal elastic membrane and loss of smooth muscle cells in the medial layer of the aortic wall, accompanied by inflammatory processes in the medial and adventitial layers, leading to enlargement that may worsen over time. The majority of AAAs involve the nearly branchless infrarenal segment of the aorta below the level of the renal arteries ( Figure 1a) and represent the focus of virtually all of the data reported in this review. The most life-threatening yet insidious complication of an AAA is acute rupture, which may be preceded by local tenderness or pain in the back, the flank, or the abdomen but not uncommonly occurs without any warning symptoms whatsoever.

Spontaneous rupture of an AAA is a catastrophic medical emergency, typically accompanied by massive blood loss and hemodynamic instability. From 2005–2012, 51,475 deaths in the United States and another 39,740 in the United Kingdom were caused by AAAs ^1^. During the same study period, however, only 35,922 patients in the U.S. and 17,253 in the UK were formally admitted to hospitals with a diagnosis of ruptured AAAs. This implies that about one-third of patients with ruptured AAAs in the U.S. and over half of those in the UK died either without reaching an emergency room or soon after their arrival.

Even when patients are able to undergo urgent intervention for ruptured AAAs, their chances of surviving the event are not good. Although it appears to have improved during each of the past five decades, the pooled operative mortality rate for ruptured AAA repair in 21,523 patients was 48% (95% confidence interval [CI] 46 to 50%) in a meta-analysis of 171 articles published on this topic from 1955–1998 ^2^. Furthermore, the risk still exceeded 50% in a population-based study of 35,637 patients who received either open or endovascular repair for ruptured AAAs in the U.S. and the UK from 2005–2010 ^3^. Women, who generally are older and tend to present with larger AAAs relative to their normal aortic diameter than men, are more likely to rupture at a slightly smaller AAA size and have a higher operative mortality rate than men when they do rupture ^4^.

## Prevalence

According to a community-based study using ultrasound scanning below the level of the superior mesenteric artery in a total of 15,792 participants with a median follow-up period of 22.5 years, it has been estimated that the lifetime risk of developing an AAA measuring ≥3.0 cm in diameter is 5.6% (95% CI 4.8 to 6.1%) at the age of 45 ^5^. The prevalence was higher at ages 55 to 64 than at ages 45 to 54 (6% versus 3.2%), in men than in women (8.2% versus 3.2%), and in current smokers (10%) than in either former smokers (6.3%) or non-smokers (2.0%). However, the incidence of rupture or elective intervention was only 1.6%, suggesting that many of the smaller AAAs never enlarged to a size considered dangerous from a clinical standpoint. Ultrasound screening studies primarily targeting men aged 65 to 75 in the U.S. and the UK have found that only 13 to 17% of detected AAAs were 4.5 to 5.4 cm in diameter and just 4.1 to 12% were ≥5.5 cm ^6, 7^.

## Treatment options

Dubost performed the first open repair of an AAA in 1951 ^8^ using a thoracoabdominal incision and an aortic homograft. The development of synthetic replacement grafts ( Figure 1b) allowed open repair to be widely adopted, with a steady reduction in the mortality rate for elective AAA repair at referral centers from about 7% in 1963–1980 to 4% in 1981–1990 and to 2% in 1991–2000 ^9^. Population-based studies have shown that operative mortality tends to be inversely related to the annual volume of open AAA repairs performed by individual surgeons. In the state of New York, for example, surgeons doing the highest volume had better mortality rates than those with the lowest volume in 1985–1987 (5.6% versus 11%) ^10^ and in 2000–2011 (3.6% versus 6.4%) ^11^. Another study of 5,972 open repairs in the U.S. Nationwide Inpatient Sample from 2003–2007 also found that high-volume surgeons had better mortality rates than either medium-volume or low-volume surgeons (3.0% versus 4.3% versus 7.5%, respectively, *p*<0.0001) irrespective of hospital volumes ^12^.

Parodi and associates first reported (in English) a transfemoral technique for endovascular aneurysm repair (EVAR) in 1991 ^13^. Working independently, Volodos first published an article (in Russian) describing transfemoral repair of a thoracic aortic aneurysm using a similar handmade endograft in 1988 ^14^. Conceived as an option for patients at severe risk for open repair, the wide availability of commercial endografts and surgeons trained to use them has now made EVAR ( Figure 1c) an appealing alternative for average-risk patients. The current preference for EVAR is reflected by two large studies that comprised nearly 200,000 patients and found that EVAR was performed for 74% of all non-ruptured AAA repairs in the U.S. in 2010 ^15^ and for 61% of those in Australia, Iceland, New Zealand, and eight European countries in 2009 ^16^. In fact, the proportion of U.S. hospitals where at least 25% of elective AAA repairs involved open surgery declined from 41% in 2007 to only 18% in 2011 ( *p*<0.001) ^17^.

## Aneurysm size

Szilagyi demonstrated 50 years ago that patients whose AAAs measured >6 cm in diameter by physical examination had a much higher 5-year survival rate after open repair than when placed under observation alone (49% versus 17%) ^18^ and that AAA ruptures caused a larger proportion of deaths than myocardial infarctions (42% versus 31%) in patients who were deemed to be medically unfit for open repair ^19^. In a recent meta-analysis of 1,514 unfit patients reported in 11 previous articles, the pooled annual rupture rate was estimated to be 3.5% for AAAs that were 5.5 to 6.0 cm in diameter, 4.1% for those that were 6.1 to 7.0 cm, and 6.3% for those that were >7.0 cm ^20^. Urgent repair was offered to only 54 (32%) of the 171 patients whose AAAs ruptured, and their operative mortality rate was 58%.

Given the longstanding consensus that large AAAs should be repaired electively in appropriate candidates, four randomized trials have been done to establish whether early intervention might be beneficial in patients with smaller AAAs. Ultrasound surveillance to detect AAA expansion was compared to open repair in the UK Small Aneurysm Trial (UKSAT) ^21– 23^ and the Aneurysm Detection and Management trial (ADAM) ^24^ and later was compared to EVAR in the Comparison of surveillance versus Aortic Endografting for Small Aneurysm Repair trial (CAESAR) ^25^ and the Positive Impact of endoVascular Options for Treating Aneurysms earLy trial (PIVOTAL) ^26^. The demographics, 30-day mortality rates, and late results of the trials are summarized in Table 1. Women collectively represented only 10% of the participants in these trials, with especially few women in the Veterans Affairs ADAM trial. The 30-day mortality rates with early intervention ranged from 0.6% in the two trials using EVAR to 2.1% for open repair in ADAM and 5.8% in UKSAT. Except for a marginally significant survival benefit for early open repair at a mean of 8 years of follow-up in UKSAT, the all-cause mortality rates for early intervention or surveillance were comparable in each of the trials. All of them concluded that close observation with periodic ultrasound scanning was as safe as either open repair or EVAR as long as the AAA was <5.5 cm in diameter (<5.0 cm in PIVOTAL).

**Table 1.** Intention-to-treat analysis of early intervention versus ultrasound surveillance for small abdominal aortic aneurysms in the randomized UKSAT ^21– 23^, ADAM ^24^, CAESAR ^25^, and PIVOTAL ^26^ trials.

Treatment strategy	Open repair versus surveillance				Endovascular repair versus surveillance	
Trial	UKSAT			ADAM	CAESAR	PIVOTAL
Randomized patients	1,090			1,136	360	728
Men	902			1,127	345	631
Women	188 (17%)			9 (0.8%)	15 (4.2%)	97 (13%)
Mean age (years)	69 ± 4			68 ± 6	68.9 ± 6.8	70.5 ± 7.8
Aneurysm diameter						
Protocol diameter	4.0–5.5 cm			4.0–5.5 cm	4.1–5.4 cm	4.0–5.0 cm
Actual mean diameter	4.6 ± 0.4 cm			4.7 ± 0.4 cm	4.7 ± 0.3 cm	4.5 ± 0.3 cm
Early intervention	563 (517 *)			569 (380 *)	182 (175 *)	366 (326 *)
Surveillance	527 (489 *)			567 (516 *)	178 (172 *)	362 (350 *)
30-day mortality rate for early intervention	5.8%			2.1% (2.7% in-hospital)	0.6%	0.6%
Follow-up period	Range 3–7 years Mean 4.6 years	Range 6–10 years Mean 8 years	12 years	Range 3.5–8 years Mean 4.9 years	Median 32.4 months (early intervention) Median 30.9 months (surveillance)	Range 0–41 months Mean 20 ± 12 months
Survival rate						
Early intervention	64%	53%	36%	75%	86%	96%
Surveillance	64%	45% *p*=0.03	33%	78%	90%	96%
Rupture rate while under surveillance	1.0% annually	3.2% annually	4.4% crude	0.6% annually	1.1% crude	0.6% crude
Men	NR	Odds ratio, 1.0 (reference set)	NR	NR	NR	NR
Women	NR	Odds ratio, 4.0 (2.0–7.9) *p*<0.001	NR	NR	NR	NR
Eventual repair						
Intervention cohort	520 (92%)	520 (92%)	528 (94%)	527 (93%)	175 (96%)	315 (86%)
Surveillance cohort	321 (61%)	327 (62%)	401 (76%)	349 (62%)	85 (48%)	112 (31%)
Surveillance outcome by aneurysm diameter						
Survival rate	4.0–4.4 cm: 75% 4.5–4.8 cm: 73% 4.9–5.5 cm: 64%	4.0–4.4 cm: 56% 4.5–4.8 cm: 54% 4.9–5.5 cm: 43%	4.0–4.4 cm: 38% 4.5–4.8 cm: 35% 4.9–5.5 cm: 26%	4.0–4.4 cm: 84% 4.5–4.9 cm: 82% 5.0–5.4 cm: 69%	NR	NR

*Patients who actually received early treatment or surveillance ADAM, Aneurysm Detection and Management trial; CAESAR, Comparison of Surveillance versus Aortic Endografting for Small Aneurysm Repair trial; NR, not reported; PIVOTAL, Positive Impact of endoVascular Options for Treating Aneurysms earLy trial; UKSAT, United Kingdom Small Aneurysm Trial.

**Figure 2.**
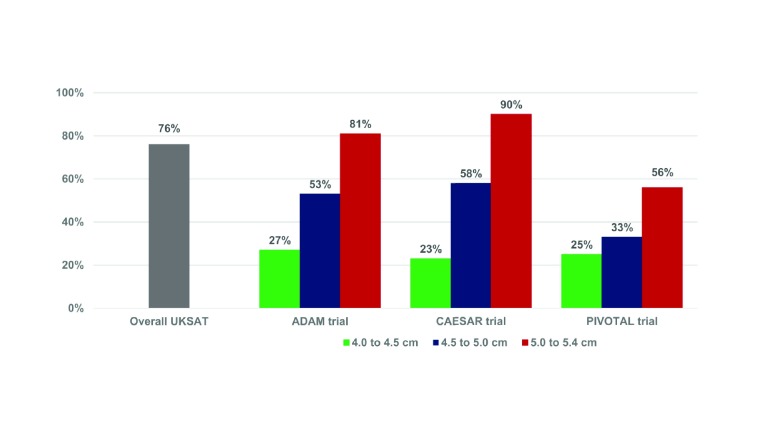
Eventual repair rates for abdominal aortic aneurysms in the surveillance cohorts of the UKSAT ^23^, ADAM ^24^, CAESAR ^25^, and PIVOTAL ^26^ trials. The reasons for the eventual repairs included rupture, the onset of back pain or local tenderness, rapid growth on consecutive ultrasound scans or enlargement to a size exceeding the trial protocol, and patient preference. ADAM, Aneurysm Detection and Management trial; CAESAR, Comparison of surveillance versus Aortic Endografting for Small Aneurysm Repair trial; PIVOTAL, Positive Impact of endoVascular Options for Treating Aneurysms earLy trial; UKSAT, United Kingdom Small Aneurysm Trial.

Two additional findings from these trials are worth mentioning. First, there was a greater risk for rupture in the 93 women than in the 434 men in the surveillance cohort of UKSAT, the only trial having a representative number of women. By a mean of 8 years, fatal ruptures caused 12 (14%) of the 85 deaths in women versus 19 (4.6%, *p*<0.001) of the 411 deaths in men. The incidence of either fatal or non-fatal ruptures also was higher among women (hazard ratio 4.0, 95% CI 2.0 to 7.9, *p*<0.001). Second, a substantial number of patients in the surveillance cohorts of all four trials eventually underwent open repair or EVAR ( Figure 2). In addition to rupture, the reasons included the onset of back pain or abdominal tenderness, patient preference, and, probably most commonly, either rapid expansion on consecutive ultrasound scans or enlargement to a size that exceeded the maximum permitted by the trial protocol. Late intervention rates correlated directly with baseline AAA diameters in ADAM, CAESAR, and PIVOTAL.

## Elective EVAR versus open repair

The advantages and liabilities of open repair and EVAR were reasonably well recognized, even before the results of most randomized trials were reported ^8^. Open repair had a higher procedural mortality rate but few late graft-related complications and a negligible risk for late AAA rupture. Conversely, EVAR had a low early mortality rate but a higher incidence of secondary endograft-related interventions, the majority being done to treat endoleaks that had continued to pressurize the aneurysm sac at endograft fixation points (type I), from retrograde flow in lumbar arteries or the inferior mesenteric artery (type II), or through endograft modular separations or fabric tears (type III). Since each might potentially cause sac expansion and eventual rupture, manufacturers have routinely recommended annual computed tomography (CT) scans to detect such problems. In an attempt to avoid the expense and radiation exposure of repeated CT scanning, however, a growing consensus among surgeons now appears to favor long-term ultrasound surveillance for patients whose initial post-EVAR CT scans show no evidence of endograft complications ^27^.

According to manufacturers’ Instructions for Use (IFU), the anatomic criteria for conventional transfemoral EVAR generally include 1) a “neck” of non-aneurysmal aorta distal to the renal arteries measuring ≥15 mm in length and <28 mm in diameter, 2) neck angulation of <60° from the center line between the lowest renal artery and the aortic bifurcation, and 3) an iliac artery diameter ranging from 8–20 mm ^28^. In 2011, an analysis of 1,063 pre-EVAR CT scans found that women were less likely than men to satisfy IFU with respect to neck length (37% versus 53%), neck angulation (74% versus 88%), and iliac diameter (42% versus 64%), with only 12% of women and 32% of men meeting all three criteria ^29^. This does not mean that EVAR cannot be adapted to many of the remaining patients, but the incidence of subsequent AAA sac expansion is higher when IFU are not followed ^28^. Anatomic EVAR limitations are particularly relevant in women, who have a greater overall risk for procedural iliac artery injuries ^30^ and late endograft limb occlusions ^31^.

Against this background, four major, non-industry sponsored randomized trials were conducted from 1999–2009 to compare the results of elective open repair and EVAR in patients who were medically and anatomically suitable for either procedure, including the EVAR-1 trial ^32– 34^, the Dutch Randomized Endovascular Aneurysm Management trial (DREAM) ^35– 38^, the Open Versus Endovascular Repair trial (OVER) ^39– 43^, and the Anevrysme de l’aorte abdominale: Chirurgie versus Endoprothese trial (ACE) ^44^. Two demographic features of these trials were different from the earlier small aneurysm trials: only 5.6% of the patients were women and the AAAs were larger, with mean diameters ranging from 5.5 cm in ACE to as much as 6.5 cm in EVAR-1. Additional demographics, the procedural risks, and long-term outcomes in the EVAR trials are presented in Table 2.

**Table 2.** Intention-to-treat analysis of open versus endovascular repair for non-ruptured abdominal aortic aneurysms in the randomized EVAR-1 ^32– 34^, DREAM ^35– 37^, OVER ^39– 40^, and ACE ^44^ trials.

Trial	EVAR-1		DREAM		OVER		ACE	
	Open	EVAR	Open	EVAR	Open	EVAR	Open	EVAR
Patients randomized	1,252		351		881		299	
Treatment allocated	626 (602 *)	626 (614 *)	178 (169 *)	173 (170 *)	437 (416 *)	444 (427 *)	149 (135 *)	150 (163 *)
Men	570	565	161	161	435	441	146	150
Women	56	61	17	12	2	6	3	0
Mean age	74.0 ± 6.1	74.0 ± 6.1	69.6 ± 6.8	70.7 ± 6.6	70.5 ± 7.8	69.6 ± 7.8	70 ± 7.1	70 ± 7.7
Aneurysm diameter								
Protocol diameter	≥5.5 cm		≥5.0 cm		≥5.0 cm		>5.0 cm in men >4.5 cm in women	
Actual mean diameter	6.5 ± 1.0 cm	6.4 ± 0.9 cm	6.0 ± 0.8 cm	6.1 ± 0.9 cm	5.7 ± 1.0 cm	5.7 ± 0.8 cm	5.6 ± 0.7 cm	5.5 ± 0.8 cm
Early outcome								
30-day mortality rate	4.3%	1.8% OR 0.39 (0.18–0.87) *p*=0.02	4.6% *	1.2% * *p*=0.10	2.3%	0.2% *p*=0.006	0.6%	1.3%
In-hospital mortality rate	6.0%	2.3% OR 0.39 (0.20–0.76) *p*=0.006	NR	NR	3.0%	0.5% *p*=0.004	NR	NR
Median hospital length of stay	12 days	7 days *p*<0.0001	NR	NR	7 days	3 days *p*<0.001	10 days	6 days *p*<0.0001
Follow-up period	Mean 12.7 ± 1.5 years		Median 6.4 years		Mean 5.2 years		Mean 2.5 ± 1.2 years Median 3 years	
All-cause mortality rate	42% (8 years) 71% (15 years)	42% (8 years) 74% (15 years)	10% (2 years) 34% (6 years)	10% (2 years) 34% (6 years)	9.8% (2 years) 33% (8 years)	7.0% (2 years) 33% (8 years)	3.5% (1 year) 13% (3 years)	4.8% (1 year) 14% (3 years)
Aneurysm- related mortality	6.4% (8 years) 7.2% (15 years)	5.8% (8 years) 8.9% (15 years)	5.7% (2 years)	2.1% (2 years)	3.0% (2 years) 3.7% (8 years)	1.4% (2 years) 2.3% (8 years)	0.6% (3 years)	4.0% (3 years)
Other events								
Aneurysm rupture	0.5% (8 years) 0.8% (15 years)	2.9% (8 years) 5.0% (15 years)	0 (2 years) 0 (6 years)	0 (2 years) 0.6% (6 years)	0 (2 years) 0 (8 years)	0.9% (2 years) 1.4% (8 years)	0 (3 years)	2.0% (3 years)
Secondary intervention	1.7% (8 years) 12% (15 years)	5.1% (8 years) 26% (15 years) HR 2.42 (1.82-3.21) *p*<0.0001	18% (6 years)	30% (6 years) *p*=0.03	13% (2 years) 18% (8 years)	14% (2 years) 22% (8 years)	2.7% (3 years)	16% (3 years) *p*<0.0001

*Patients who actually received open or endovascular repair. ACE, Anevrysme de l’aorte abdominale: Chirurgie versus Endoprothese trial; DREAM, Dutch Randomized Endovascular Aneurysm Management trial; EVAR, endovascular aneurysm repair; HR, hazard ratio; NR, not reported; OR, odds ratio; OVER, Veterans Affairs Open versus Endovascular Repair trial.

The two largest trials found that EVAR had a significant advantage over open repair with respect to the 30-day (1.8% versus 4.3% in EVAR-1, *p*=0.02; 0.2% versus 2.3% in OVER, *p*=0.006) and the in-hospital (2.3% versus 6.0% in EVAR-1, *p*=0.006; 0.5% versus 3.0% in OVER, *p*=0.004) mortality rates. EVAR also was associated with significantly shorter median lengths of stay in the hospital than open repair in EVAR-1 (7 versus 12 days, *p*<0.0001), in OVER (3 versus 7 days, *p*<0.001), and in ACE (6 versus 10 days, *p*<0.0001). The influence of the early survival benefit for EVAR on all-cause mortality rates usually lasted for about 3 years, after which other common causes of death again took precedence. Aneurysm-related mortality, which included the initial procedural deaths as well as the subsequent fatal ruptures, did not shift in favor of open repair until late in follow-up. However, the secondary intervention rates were substantially higher after EVAR than after open repair at all periods of observation in most of the trials.

**Figure 3.**
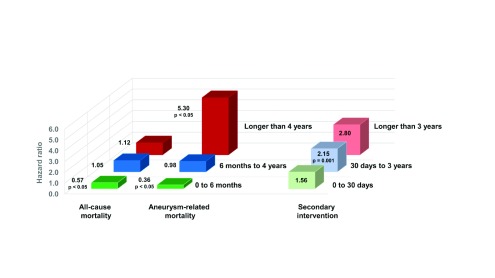
Hazard ratios for endovascular aortic aneurysm repair versus open repair in a meta-analysis of pooled individual patient data from the EVAR-1, DREAM, OVER, and ACE trials ^45^. EVAR had a survival advantage at 6 months because of a lower 30-day mortality rate, followed by a gradually higher incidence of aneurysm-related deaths or re-interventions. ACE, Anevrysme de l’aorte abdominale: Chirurgie versus Endoprothese trial; DREAM, Dutch Randomized Endovascular Aneurysm Management trial; EVAR, endovascular aneurysm repair; OVER, Open Versus Endovascular Repair trial.

A meta-analysis of pooled individual patient data adds further perspective regarding the timing of certain outcomes of interest at a median follow-up of 5.5 years for 2,783 patients in EVAR-1, DREAM, OVER, and ACE ^45^. As shown in Figure 3, the hazard ratios (HRs) for all-cause and aneurysm-related mortality were significantly lower among patients allocated to EVAR during the first 6 months following their randomization. There then was a gradual reversal in the HRs, which was inconsequential for all-cause mortality but eventually revealed a significantly higher aneurysm-related mortality in EVAR patients at longer than 4 years of follow-up (HR 5.30, 95% CI 1.52 to 18.46, *p*<0.05). Meanwhile, the HRs for secondary intervention were higher among EVAR patients even within the first month, attained statistical significance at 30 days to 3 years ( *p*<0.05), and peaked at longer than 3 years (HR 2.80, 95% CI 1.85 to 4.24).

The OVER trialists have reported a detailed analysis of endoleaks, 32% of which were treated by re-interventions ^41^. During a mean follow-up of approximately 6 years, endoleaks were identified in 30% of patients after successful EVAR, 76% of the endoleaks being classified as type II. Type II endoleaks were likely to be associated with AAA expansion when discovered later than 1 year after EVAR ( *p*<0.0001), though 84% of all type II endoleaks were detected earlier and 60% of them resolved spontaneously. Despite the expense of CT scan surveillance and re-interventions, OVER still found that EVAR was cost effective compared to open repair ^42, 43^. This was not the case in EVAR-1 ^33^, in DREAM ^38^, or in Markov models derived from all four trials ^46^.

Finally, it must be mentioned for completeness that a second randomized trial, the EVAR-2 trial, also was conducted in the UK in order to compare EVAR to observation alone in 404 patients who had AAAs measuring ≥5.5 cm in diameter (mean 6.8 cm) but were medically unfit for open repair and thus could not be enrolled in EVAR-1 ^47, 48^. The 30-day mortality rate for EVAR was 7.3%, and fatal ruptures occurred in 31% of the patients in the observation cohort during a median follow-up of 3.1 years. The aneurysm-related mortality was lower with EVAR (HR 0.53, 95% CI 0.32 to 0.89, *p*=0.02), but this was not associated with a significant benefit for EVAR in terms of all-cause mortality (HR 0.99, 95% CI 0.78 to 1.27, *p*=0.97). Endograft-related complications occurred in 48% of EVAR patients, 27% of whom were treated with secondary interventions during the first 6 years of surveillance. This trial concluded that EVAR did not improve long-term survival in patients having serious medical comorbidities and, of course, that it was more costly than observation.

## Ruptured aneurysm repair

Few randomized trials have been done to compare open repair to EVAR in patients with ruptured AAAs, largely because it is so critically important not to delay definitive treatment. There simply might not be enough time to perform the imaging studies that are necessary to determine whether many hemodynamically unstable patients are anatomically eligible for EVAR as well as for open repair. Conceding the potential for bias in this regard, a meta-analysis of the individual data for a total of 836 patients in three randomized trials has reported pooled 30-day mortality rates of 31% in patients allocated to EVAR versus 34% in those receiving open repair ^49^. Early mortality rates also were closely comparable at 90 days (34% and 38%, respectively), with a modest advantage for EVAR only among the 160 women in the three trials.

Using a risk stratification system based on age >76 years, preoperative cardiac arrest or loss of consciousness, and the necessity for suprarenal aortic clamping during open procedures, a study from a large registry maintained by the Society for Vascular Surgery has attempted to clarify the relative benefits of open repair and EVAR in 1,165 patients who underwent ruptured AAA repair from 2003–2013 ^50^. Open repair was done in 514 of these patients and EVAR in 651, with EVAR having a lower in-hospital mortality rate (25% versus 33%, *p*=0.001). The mortality advantage for EVAR was most evident in medium-risk patients (37% versus 48%, *p*=0.02) and trended towards significance in low-risk patients (10% versus 15%, *p*=0.07). However, EVAR was not associated with any mortality benefit in high-risk patients (95% versus 79%, *p*=0.17). Unfortunately, a truly all-inclusive randomized trial to resolve these issues may never be feasible.

## Medicare correlations

Population-based data help to translate the findings of randomized trials into a real-world setting. Information for a total of 128,598 Medicare patients across the U.S. confirms that the proportion of elective AAA repairs performed using EVAR instead of open procedures increased from 36% in 2001 ^51^ to 82% in 2008 ^52^. During that time, death occurred within 30 days or during the index hospital admission in 1.6% of propensity-matched patients who had EVAR versus 5.2% of those who had open repair (relative risk for open repair 3.22, 95% CI 2.95 to 3.51, *p*<0.001). EVAR patients also sustained fewer medical complications and had shorter median lengths of stay (2 versus 7 days, *p*<0.001). Similar trends have been reported among 23,670 patients of all ages who had EVAR or open repair for non-ruptured AAAs in the state of California from 2001–2009 ^53^.

The procedural mortality benefit with EVAR lasted for 3 years in propensity-matched cohorts, after which all-cause mortality rates for EVAR and open repair converged both at 5 years (each 34%) and at 8 years (each 55%). The rupture rate was higher at 8 years after EVAR (5.4% versus 1.4%, *p*<0.001), and EVAR patients also had a higher incidence of device-related re-interventions (19% versus 3.7%, *p*<0.001). Re-interventions for abdominal wall hernias or the lysis of intra-abdominal adhesions were more common after open repair (18% versus 8.2%, *p*<0.001) and so were hospital admissions for the conservative management of intestinal obstruction (22% versus 17%, *p*<0.001). Nevertheless, some of these were not necessarily related specifically to the previous AAA repair.

## Conclusions

Readers should also be made aware that great progress is taking place at dedicated aortic centers with the use of fenestrated and branched endografts to repair aortic aneurysms extending above the renal arteries ^54, 55^, but EVAR already has become a valuable and widely available option for appropriate infrarenal AAAs. Size is the primary factor determining whether any intervention is necessary. Provided there are no compelling medical contraindications, early elective treatment is preferred for AAAs that are ≥5.5 cm in diameter and may deserve consideration for slightly smaller AAAs in young, otherwise-healthy patients, particularly in women. EVAR has distinct short-term advantages in eligible candidates, but it requires lifelong surveillance, has a higher aneurysm-related re-intervention rate, and is associated with a low but measurable risk for late rupture.

## Abbreviations

AAA, abdominal aortic aneurysm; ACE, Anevrysme de l’aorte abdominale: Chirurgie versus Endoprothese trial; ADAM, Aneurysm Detection and Management trial; CAESAR, Comparison of surveillance versus Aortic Endografting for Small Aneurysm Repair trial; CI, confidence interval; CT, computed tomography; DREAM, Dutch Randomized Endovascular Aneurysm Management trial; EVAR, endovascular aneurysm repair; HR, hazard ratio; IFU, Instructions for Use; OVER, Open Versus Endovascular Repair trial; PIVOTAL, Positive Impact of endoVascular Options for Treating Aneurysms earLy trial; UKSAT, United Kingdom Small Aneurysm Trial.
